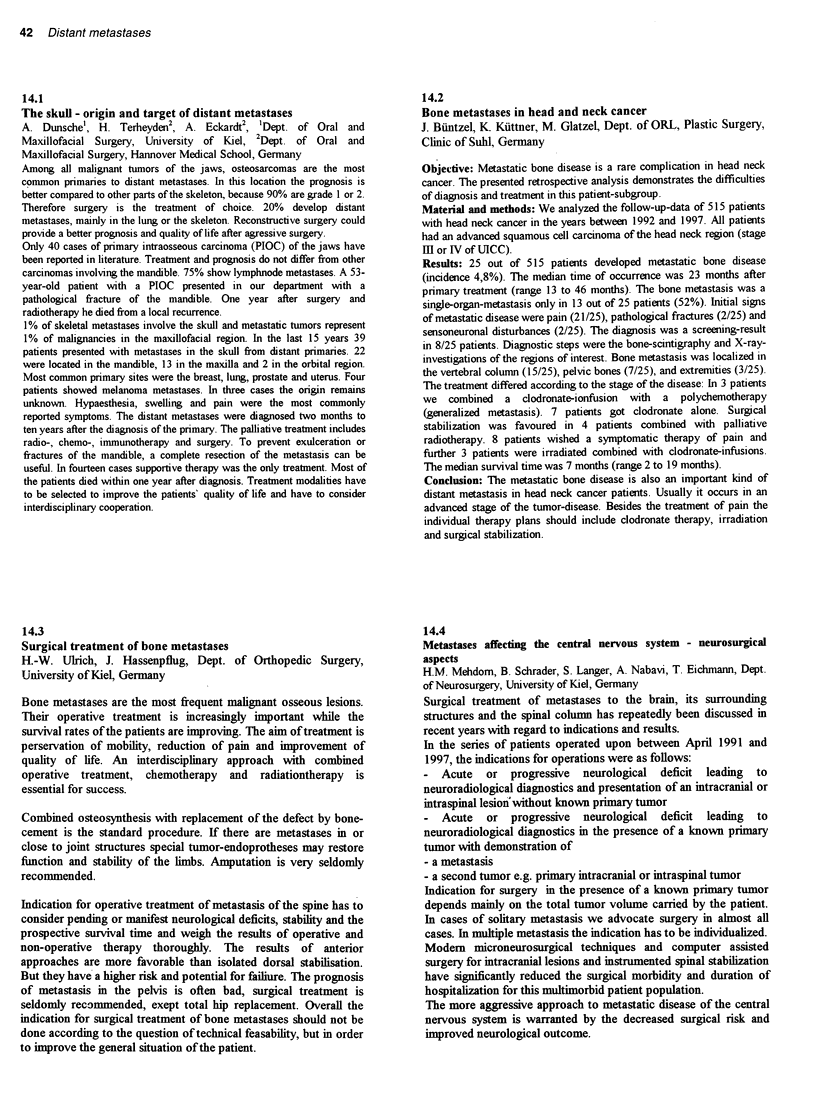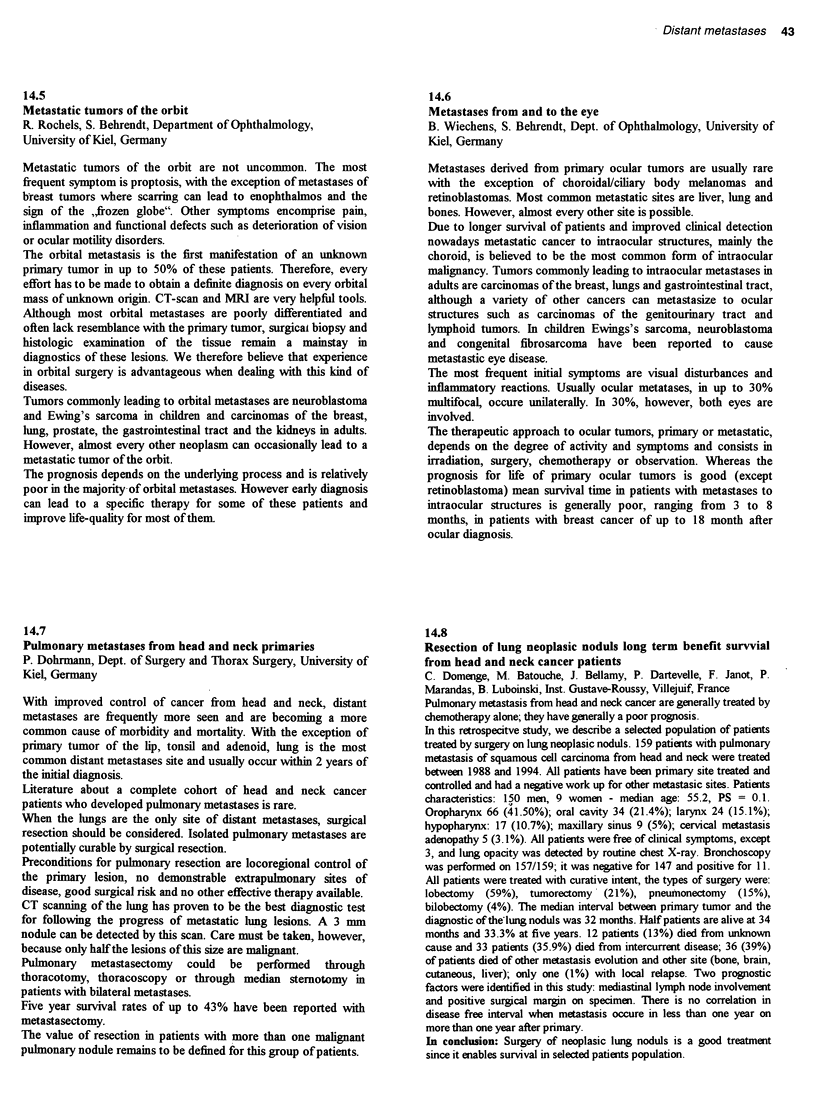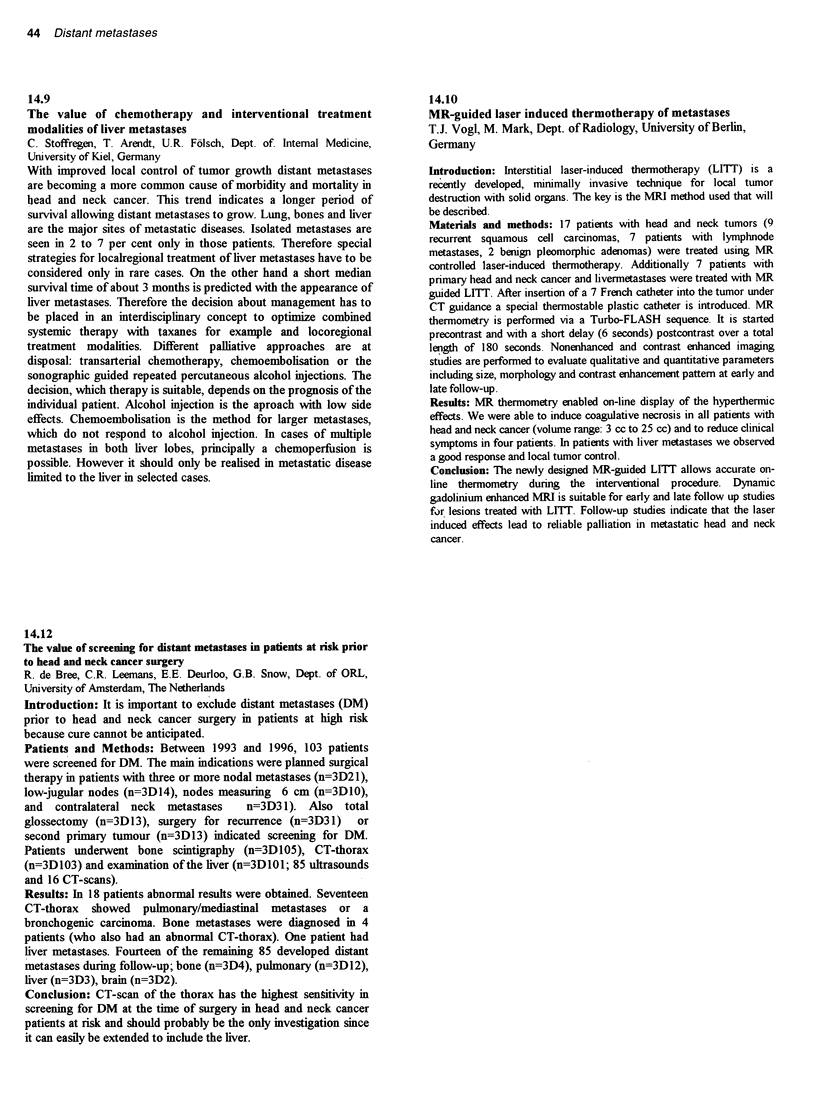# Distant metastases

**Published:** 1998

**Authors:** 


### 14.1
### The skull - origin and target of distant metastases

A. Dunsche[1], H. Terheyden[2], A. Eckardt[2], [1]Dept. of Oral and Maxillofacial Surgery, University of Kiel, [2]Dept. of Oral and Maxillofacial Surgery, Hannover Medical School, Germany

Among all malignant tumors of the jaws, osteosarcomas are the most common primaries to distant metastases. In this location the prognosis is better compared to other parts of the skeleton, because 90% are grade 1 or 2. Therefore surgery is the treatment of choice. 20% develop distant metastases, mainly in the lung or the skeleton. Reconstructive surgery could provide a better prognosis and quality of life after agressive surgery.

Only 40 cases of primary intraosseous carcinoma (PIOC) of the jaws have been reported in literature. Treatment and prognosis do not differ from other carcinomas involving the mandible. 75% show lymphnode metastases. A 53-year-old patient with a PIOC presented in our department with a pathological fracture of the mandible. One year after surgery and radiotherapy he died from a local recurrence.

1% of skeletal metastases involve the skull and metastatic tumors represent 1% of malignancies in the maxillofacial region. In the last 15 years 39 patients presented with metastases in the skull from distant primaries. 22 were located in the mandible, 13 in the maxilla and 2 in the orbital region. Most common primary sites were the breast, lung, prostate and uterus. Four patients showed melanoma metastases. In three cases the origin remains unknown. Hypaesthesia, swelling and pain were the most commonly reported symptoms. The distant metastases were diagnosed two months to ten years after the diagnosis of the primary. The palliative treatment includes radio-, chemo-, immunotherapy and surgery. To prevent exulceration or fractures of the mandible, a complete resection of the metastasis can be useful. In fourteen cases supportive therapy was the only treatment. Most of the patients died within one year after diagnosis. Treatment modalities have to be selected to improve the patients' quality of life and have to consider interdisciplinary cooperation.

### 14.2
### Bone metastases in head and neck cancer

J. Büntzel, K. Küttner, M. Glatzel, Dept. of ORL, Plastic Surgery, Clinic of Suhl, Germany

**Objective:** Metastatic bone disease is a rare complication in head neck cancer. The presented retrospective analysis demonstrates the difficulties of diagnosis and treatment in this patient-subgroup.

**Material and methods:** We analyzed the follow-up-data of 515 patients with head neck cancer in the years between 1992 and 1997. All patients had an advanced squamous cell carcinoma of the head neck region (stage III or IV of UICC).

**Results:** 25 out of 515 patients developed metastatic bone disease (incidence 4,8%). The median time of occurrence was 23 months after primary treatment (range 13 to 46 months). The bone metastasis was a single-organ-metastasis only in 13 out of 25 patients (52%). Initial signs of metastatic disease were pain (21/25), pathological fractures (2/25) and sensoneuronal disturbances (2/25). The diagnosis was a screening-result in 8/25 patients. Diagnostic steps were the bone-scintigraphy and X-ray-investigations of the regions of interest. Bone metastasis was localized in the vertebral column (15/25), pelvic bones (7/25), and extremities (3/25). The treatment differed according to the stage of the disease: In 3 patients we combined a clodronate-ionfusion with a polychemotherapy (generalized metastasis). 7 patients got clodronate alone. Surgical stabilization was favoured in 4 patients combined with palliative radiotherapy. 8 patients wished a symptomatic therapy of pain and further 3 patients were irradiated combined with clodronate-infusions. The median survival time was 7 months (range 2 to 19 months).

**Conclusion:** The metastatic bone disease is also an important kind of distant metastasis in head neck cancer patients. Usually it occurs in an advanced stage of the tumor-disease. Besides the treatment of pain the individual therapy plans should include clodronate therapy, irradiation and surgical stabilization.

### 14.3
### Surgical treatment of bone metastases

H.-W. Ulrich, J. Hassenpflug, Dept. of Orthopedic Surgery, University of Kiel, Germany

Bone metastases are the most frequent malignant osseous lesions. Their operative treatment is increasingly important while the survival rates of the patients are improving. The aim of treatment is perservation of mobility, reduction of pain and improvement of quality of life. An interdisciplinary approach with combined operative treatment, chemotherapy and radiationtherapy is essential for success.

Combined osteosynthesis with replacement of the defect by bone-cement is the standard procedure. If there are metastases in or close to joint structures special tumor-endoprotheses may restore function and stability of the limbs. Amputation is very seldomly recommended.

Indication for operative treatment of metastasis of the spine has to consider pending or manifest neurological deficits, stability and the prospective survival time and weigh the results of operative and non-operative therapy thoroughly. The results of anterior approaches are more favorable than isolated dorsal stabilisation. But they have a higher risk and potential for failiure. The prognosis of metastasis in the pelvis is often bad, surgical treatment is seldomly recommended, exept total hip replacement. Overall the indication for surgical treatment of bone metastases should not be done according to the question of technical feasability, but in order to improve the general situation of the patient.

### 14.4
### Metastases affecting the central nervous system - neurosurgical aspects

H.M. Mehdorn, B. Schrader, S. Langer, A. Nabavi, T. Eichmann, Dept. of Neurosurgery, University of Kiel, Germany

Surgical treatment of metastases to the brain, its surrounding structures and the spinal column has repeatedly been discussed in recent years with regard to indications and results.

In the series of patients operated upon between April 1991 and 1997, the indications for operations were as follows:

- Acute or progressive neurological deficit leading to neuroradiological diagnostics and presentation of an intracranial or intraspinal lesion without known primary tumor
- Acute or progressive neurological deficit leading to neuroradiological diagnostics in the presence of a known primary tumor with demonstration of
- a metastasis
- a second tumor e.g. primary intracranial or intraspinal tumor

Indication for surgery in the presence of a known primary tumor depends mainly on the total tumor volume carried by the patient. In cases of solitary metastasis we advocate surgery in almost all cases. In multiple metastasis the indication has to be individualized. Modern microneurosurgical techniques and computer assisted surgery for intracranial lesions and instrumented spinal stabilization have significantly reduced the surgical morbidity and duration of hospitalization for this multimorbid patient population.

The more aggressive approach to metastatic disease of the central nervous system is warranted by the decreased surgical risk and improved neurological outcome.

### 14.5
### Metastatic tumors of the orbit

R. Rochels, S. Behrendt, Department of Ophthalmology, University of Kiel, Germany

Metastatic tumors of the orbit are not uncommon. The most frequent symptom is proptosis, with the exception of metastases of breast tumors where scarring can lead to enophthalmos and the sign of the „frozen globe". Other symptoms encomprise pain, inflammation and functional defects such as deterioration of vision or ocular motility disorders.
The orbital metastasis is the first manifestation of an unknown primary tumor in up to 50% of these patients. Therefore, every effort has to be made to obtain a definite diagnosis on every orbital mass of unknown origin. CT-scan and MRI are very helpful tools. Although most orbital metastases are poorly differentiated and often lack resemblance with the primary tumor, surgical biopsy and histologic examination of the tissue remain a mainstay in diagnostics of these lesions. We therefore believe that experience in orbital surgery is advantageous when dealing with this kind of diseases.
Tumors commonly leading to orbital metastases are neuroblastoma and Ewing's sarcoma in children and carcinomas of the breast, lung, prostate, the gastrointestinal tract and the kidneys in adults. However, almost every other neoplasm can occasionally lead to a metastatic tumor of the orbit.
The prognosis depends on the underlying process and is relatively poor in the majority of orbital metastases. However early diagnosis can lead to a specific therapy for some of these patients and improve life-quality for most of them.

### 14.6
### Metastases from and to the eye

B. Wiechens, S. Behrendt, Dept. of Ophthalmology, University of Kiel, Germany

Metastases derived from primary ocular tumors are usually rare with the exception of choroidal/ciliary body melanomas and retinoblastomas. Most common metastatic sites are liver, lung and bones. However, almost every other site is possible.
Due to longer survival of patients and improved clinical detection nowadays metastatic cancer to intraocular structures, mainly the choroid, is believed to be the most common form of intraocular malignancy. Tumors commonly leading to intraocular metastases in adults are carcinomas of the breast, lungs and gastrointestinal tract, although a variety of other cancers can metastasize to ocular structures such as carcinomas of the genitourinary tract and lymphoid tumors. In children Ewings's sarcoma, neuroblastoma and congenital fibrosarcoma have been reported to cause metastastic eye disease.
The most frequent initial symptoms are visual disturbances and inflammatory reactions. Usually ocular metatases, in up to 30% multifocal, occure unilaterally. In 30%, however, both eyes are involved.
The therapeutic approach to ocular tumors, primary or metastatic, depends on the degree of activity and symptoms and consists in irradiation, surgery, chemotherapy or observation. Whereas the prognosis for life of primary ocular tumors is good (except retinoblastoma) mean survival time in patients with metastases to intraocular structures is generally poor, ranging from 3 to 8 months, in patients with breast cancer of up to 18 month after ocular diagnosis.

### 14.7
### Pulmonary metastases from head and neck primaries

P. Dohrmann, Dept. of Surgery and Thorax Surgery, University of Kiel, Germany

With improved control of cancer from head and neck, distant metastases are frequently more seen and are becoming a more common cause of morbidity and mortality. With the exception of primary tumor of the lip, tonsil and adenoid, lung is the most common distant metastases site and usually occur within 2 years of the initial diagnosis.
Literature about a complete cohort of head and neck cancer patients who developed pulmonary metastases is rare.
When the lungs are the only site of distant metastases, surgical resection should be considered. Isolated pulmonary metastases are potentially curable by surgical resection.
Preconditions for pulmonary resection are locoregional control of the primary lesion, no demonstrable extrapulmonary sites of disease, good surgical risk and no other effective therapy available.
CT scanning of the lung has proven to be the best diagnostic test for following the progress of metastatic lung lesions. A 3 mm nodule can be detected by this scan. Care must be taken, however, because only half the lesions of this size are malignant.
Pulmonary metastasectomy could be performed through thoracotomy, thoracoscopy or through median sternotomy in patients with bilateral metastases.
Five year survival rates of up to 43% have been reported with metastasectomy.
The value of resection in patients with more than one malignant pulmonary nodule remains to be defined for this group of patients.

### 14.8
### Resection of lung neoplasic noduls long term benefit survvial from head and neck cancer patients

C. Domenge, M. Batouche, J. Bellamy, P. Dartevelle, F. Janot, P. Marandas, B. Luboinski, Inst. Gustave-Roussy, Villejuif, France

Pulmonary metastasis from head and neck cancer are generally treated by chemotherapy alone; they have generally a poor prognosis.
In this retrospecitve study, we describe a selected population of patients treated by surgery on lung neoplasic noduls. 159 patients with pulmonary metastasis of squamous cell carcinoma from head and neck were treated between 1988 and 1994. All patients have been primary site treated and controlled and had a negative work up for other metastasic sites. Patients characteristics: 150 men, 9 women - median age: 55.2, PS = 0.1. Oropharynx 66 (41.50%); oral cavity 34 (21.4%); larynx 24 (15.1%); hypopharynx: 17 (10.7%); maxillary sinus 9 (5%); cervical metastasis adenopathy 5 (3.1%). All patients were free of clinical symptoms, except 3, and lung opacity was detected by routine chest X-ray. Bronchoscopy was performed on 157/159; it was negative for 147 and positive for 11. All patients were treated with curative intent, the types of surgery were: lobectomy (59%), tumorectomy (21%), pneumonectomy (15%), bilobectomy (4%). The median interval between primary tumor and the diagnostic of the lung noduls was 32 months. Half patients are alive at 34 months and 33.3% at five years. 12 patients (13%) died from unknown cause and 33 patients (35.9%) died from intercurrent disease; 36 (39%) of patients died of other metastasis evolution and other site (bone, brain, cutaneous, liver); only one (1%) with local relapse. Two prognostic factors were identified in this study: mediastinal lymph node involvement and positive surgical margin on specimen. There is no correlation in disease free interval when metastasis occure in less than one year on more than one year after primary.
**In conclusion:** Surgery of neoplasic lung noduls is a good treatment since it enables survival in selected patients population.

### 14.9

**The value of chemotherapy and interventional treatment modalities of liver metastases**

C. Stoffregen, T. Arendt, U.R. Fölsch, Dept. of. Internal Medicine, University of Kiel, Germany

With improved local control of tumor growth distant metastases are becoming a more common cause of morbidity and mortality in head and neck cancer. This trend indicates a longer period of survival allowing distant metastases to grow. Lung, bones and liver are the major sites of metastatic diseases. Isolated metastases are seen in 2 to 7 per cent only in those patients. Therefore special strategies for localregional treatment of liver metastases have to be considered only in rare cases. On the other hand a short median survival time of about 3 months is predicted with the appearance of liver metastases. Therefore the decision about management has to be placed in an interdisciplinary concept to optimize combined systemic therapy with taxanes for example and locoregional treatment modalities. Different palliative approaches are at disposal: transarterial chemotherapy, chemoembolisation or the sonographic guided repeated percutaneous alcohol injections. The decision, which therapy is suitable, depends on the prognosis of the individual patient. Alcohol injection is the aproach with low side effects. Chemoembolisation is the method for larger metastases, which do not respond to alcohol injection. In cases of multiple metastases in both liver lobes, principally a chemoperfusion is possible. However it should only be realised in metastatic disease limited to the liver in selected cases.

### 14.10

**MR-guided laser induced thermotherapy of metastases**

T.J. Vogl, M. Mark, Dept. of Radiology, University of Berlin, Germany

**Introduction:** Interstitial laser-induced thermotherapy (LITT) is a recently developed, minimally invasive technique for local tumor destruction with solid organs. The key is the MRI method used that will be described.

**Materials and methods:** 17 patients with head and neck tumors (9 recurrent squamous cell carcinomas, 7 patients with lymphnode metastases, 2 benign pleomorphic adenomas) were treated using MR controlled laser-induced thermotherapy. Additionally 7 patients with primary head and neck cancer and livermetastases were treated with MR guided LITT. After insertion of a 7 French catheter into the tumor under CT guidance a special thermostable plastic catheter is introduced. MR thermometry is performed via a Turbo-FLASH sequence. It is started precontrast and with a short delay (6 seconds) postcontrast over a total length of 180 seconds. Nonenhanced and contrast enhanced imaging studies are performed to evaluate qualitative and quantitative parameters including size, morphology and contrast enhancement pattern at early and late follow-up.

**Results:** MR thermometry enabled on-line display of the hyperthermic effects. We were able to induce coagulative necrosis in all patients with head and neck cancer (volume range: 3 cc to 25 cc) and to reduce clinical symptoms in four patients. In patients with liver metastases we observed a good response and local tumor control.

**Conclusion:** The newly designed MR-guided LITT allows accurate on-line thermometry during the interventional procedure. Dynamic gadolinium enhanced MRI is suitable for early and late follow up studies for lesions treated with LITT. Follow-up studies indicate that the laser induced effects lead to reliable palliation in metastatic head and neck cancer.

### 14.12

**The value of screening for distant metastases in patients at risk prior to head and neck cancer surgery**

R. de Bree, C.R. Leemans, E.E. Deurloo, G.B. Snow, Dept. of ORL, University of Amsterdam, The Netherlands

**Introduction:** It is important to exclude distant metastases (DM) prior to head and neck cancer surgery in patients at high risk because cure cannot be anticipated.

**Patients and Methods:** Between 1993 and 1996, 103 patients were screened for DM. The main indications were planned surgical therapy in patients with three or more nodal metastases (n=3D21), low-jugular nodes (n=3D14), nodes measuring 6 cm (n=3D10), and contralateral neck metastases n=3D31). Also total glossectomy (n=3D13), surgery for recurrence (n=3D31) or second primary tumour (n=3D13) indicated screening for DM. Patients underwent bone scintigraphy (n=3D105), CT-thorax (n=3D103) and examination of the liver (n=3D101; 85 ultrasounds and 16 CT-scans).

**Results:** In 18 patients abnormal results were obtained. Seventeen CT-thorax showed pulmonary/mediastinal metastases or a bronchogenic carcinoma. Bone metastases were diagnosed in 4 patients (who also had an abnormal CT-thorax). One patient had liver metastases. Fourteen of the remaining 85 developed distant metastases during follow-up; bone (n=3D4), pulmonary (n=3D12), liver (n=3D3), brain (n=3D2).

**Conclusion:** CT-scan of the thorax has the highest sensitivity in screening for DM at the time of surgery in head and neck cancer patients at risk and should probably be the only investigation since it can easily be extended to include the liver.